# Bayesian optimization for conformer generation

**DOI:** 10.1186/s13321-019-0354-7

**Published:** 2019-05-21

**Authors:** Lucian Chan, Geoffrey R. Hutchison, Garrett M. Morris

**Affiliations:** 1 Department of Statistics, University of Oxford, 24-29 St Giles’, Oxford, OX1 3LB UK; 2 Department of Chemistry and Chemical Engineering, University of Pittsburgh, 219 Parkman Avenue, Pittsburgh, PA 15260 USA

**Keywords:** Bayesian optimization, Gaussian processes, Conformer generation, Rotatable bond, Torsion angle, Conformational space, Molecular energetics

## Abstract

Generating low-energy molecular conformers is a key task for many areas of computational chemistry, molecular modeling and cheminformatics. Most current conformer generation methods primarily focus on generating geometrically diverse conformers rather than finding the most probable or energetically lowest minima. Here, we present a new stochastic search method called the Bayesian optimization algorithm (BOA) for finding the lowest energy conformation of a given molecule. We compare BOA with uniform random search, and systematic search as implemented in Confab, to determine which method finds the lowest energy. Energetic difference, root-mean-square deviation, and torsion fingerprint deviation are used to quantify the performance of the conformer search algorithms. In general, we find BOA requires far fewer evaluations than systematic or uniform random search to find low-energy minima. For molecules with four or more rotatable bonds, Confab typically evaluates $10^{4}$ (median) conformers in its search, while BOA only requires $10^{2}$ energy evaluations to find top candidates. Despite using evaluating fewer conformers, 20–40% of the time BOA finds lower-energy conformations than a systematic Confab search for molecules with four or more rotatable bonds.

**Electronic supplementary material:**

The online version of this article (10.1186/s13321-019-0354-7) contains supplementary material, which is available to authorized users.

## Background

Most small molecules are flexible and can adopt multiple energetically-accessible conformations. Even in medium-sized molecules, e.g. molecules with six or more rotatable bonds, there may be thousands or millions of possibilities. The multi-dimensional energy landscape and presence of huge numbers of local minima make finding low-energy conformations to be one of the key challenges in molecular modeling and cheminformatics [1–3].

A variety of tools have been developed to generate conformers, including BALLOON [4, 5], Confab [6], FROG2 [7], MOE [8], OMEGA [9] and RDKit [10]. The search algorithms implemented in these tools can be broadly classified as either systematic or stochastic. A systematic method deterministically enumerates all of the allowed torsion angles for each rotatable bond in the molecule, and always outputs the same conformer with the lowest energy. This approach is restricted to molecules with a small number of rotatable bonds because of the combinatorial explosion of states as more search dimensions are added. Stochastic methods such as Monte Carlo simulated annealing [11, 12], distance geometry [13] and genetic algorithms [14, 15], sample random values for the torsion angles, sometimes restricted to predefined ranges. Since the method is dependent on random numbers, the output conformation may vary, but they permit problems with higher dimensions to be handled.

Knowledge-based methods (e.g. [16, 17]) use predefined libraries (e.g. [18]) for torsion angles and ring conformations, and these libraries are typically created from experimentally determined structures in databases such as the Cambridge Structural Database (CSD) [19] or the Protein Data Bank (PDB) [20].

Knowledge-based methods are usually combined with different search algorithms mentioned before. For example, Experimental-Torsion Distance Geometry with basic Knowledge (ETKDG) [21] is a relatively recent algorithm implemented in RDKit that combines knowledge about preferred torsion angles with distance geometry to produce more realistic conformations.

These algorithms primarily focus on generating geometrically diverse, low-energy conformers, which are important to many applications including structure-based virtual screening, pharmacophore modeling, and generating 3D quantitative structure-activity relationships (QSAR). In this paper, we mainly focus on finding the lowest-energy conformation of a molecule instead of achieving geometric diversity. The energy landscape is effectively unknown a priori, without exhaustive—and sometimes expensive—energy evaluation. Therefore, intelligent search strategies are needed to find the lowest energy state, and its associated conformation, in the shortest time possible. Most methods perform well if the number of rotatable bonds is small, typically four or fewer. The combinatorial explosion that arises with more flexible molecules, however, makes finding this global optimum increasingly more challenging.

We present a new approach to solve this difficult search problem, namely the Bayesian optimization algorithm, or BOA. This technique learns the most likely dihedral angles for an arbitrary molecule by ‘intelligently’ sampling new conformers from the multi-dimensional potential energy surface, regardless of the energy function used.

## Bayesian optimization

The Bayesian optimization algorithm (BOA) is a particularly effective strategy to find the optimum value of objective functions that are expensive to evaluate, for instance tuning hyperparameters in machine learning models [22] and combinatorial optimization [23, 24]. BOA is applicable in situations where we do not have a closed-form expression of the objective function, but we are able to obtain observations (possibly noisy) of this function at specifically sampled values. It is particularly useful when the objective is non-convex or derivatives are not available. Moreover, BOA allows one to incorporate prior beliefs about solutions to the problem (e.g. expected dihedral angles). Accurate priors can speed a search by directing to most likely configurations. Bayesian optimization algorithm has been applied successfully in different areas in chemistry, for instance material design [25–27] and high-throughput virtual screening [28].

The general idea of BOA is to construct an approximate surrogate model of the objective function, *f*(*x*), and then exploit the model to make decisions about the next points for evaluation. Different acquisition strategies can favor exploration of the parameter space (i.e. to find more diverse conformers) or exploitation (i.e. to find local optima). BOA uses all of the information available from previous evaluations of *f*(*x*) and hence results in a procedure that can find the optimum value of a non-convex function with a relatively small number of evaluations. The general procedure of BOA is shown in Algorithm 1.

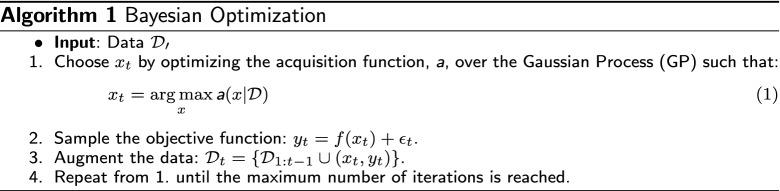



There are two major choices that must be made in the optimization procedure, namely the prior over the functions, and the acquisition function. The prior expresses assumptions or gives information about the function being optimized, while the acquisition function is used to determine the next most favorable point for evaluation, most likely to reduce uncertainty in the function’s possible values. In this section, we briefly review the general Bayesian optimization algorithm, before discussing our application to optimize conformer geometry. For an overview of the Bayesian optimization formalism and a review of previous work, see Brochu et al. [29] and Shahriari et al. [23].

### Bayesian optimization with Gaussian process priors

Different probabilistic models can be used in Bayesian optimization algorithm, for instance Gaussian process (GP) [30], random forests [24], or Student-*t* processes [31]. Gaussian processes are the default choice because of their flexibility and tractability. A GP is a stochastic process for which any finite combination of random variables follow a multivariate Gaussian distribution and its properties are determined by a mean function and a positive definite covariance function. The properties of the Gaussian distribution allow us to compute marginals and conditionals in a closed form [32].

### Covariance function

The choice of covariance function for the Gaussian process is crucial as it determines the smoothness properties of the samples. Commonly used kernels include the squared-exponential, also known as the radial basis function or RBF kernel, $k_{SE}$; and the periodic kernel, $k_{PER}$, as shown in Eqs. 2 and 3:

2 $$k_{SE}(x,y) = \sigma ^{2}\exp \left( \frac{-|x-y|^{2}}{2l^{2}}\right)$$

3 $$k_{PER}(x,y) = \sigma ^{2}\exp \left( \frac{-2\sin ^{2}(\pi |x-y|/p)}{l^2}\right)$$

where *l*, *p*, and $\sigma ^{2}$ are the length scale, period, and variance respectively.

### Acquisition function

Acquisition functions help determine which points in the search space should be evaluated, ideally providing information on the optimum value of *f*. A good acquisition function has to balance *exploration* against *exploitation*, with the trade-off based on the estimated uncertainty from a GP model. Exploration in this context involves seeking locations with high posterior variance (i.e., sampling uncertain areas), while exploitation focuses on seeking locations with low posterior mean (i.e., finding a local optimum). Three acquisition functions are commonly used, namely: probability of improvement (PI), expected improvement (EI), and GP lower confidence bound (GP-LCB), as shown in Eqs. 4, 5, and 6. The GP-LCB is also sometimes referred as upper confidence bound (UCB), when the optimization involves function maximization rather than minimization [29].

4 $$\begin{aligned} \text {PI}(x) = \Phi (z(x)) \end{aligned}$$

5 $$\begin{aligned} \text {EI}(x) = \sigma (x)(z(x)\Phi (z(x)) + \phi (z(x)) \end{aligned}$$

6 $$\begin{aligned} \text {GP-LCB}(x) = \mu (x) - \kappa \sigma (x) \end{aligned}$$

Here, $z(x) = \frac{f(x_{best})-\mu (x)}{\sigma (x)}$, where $x_{best}$, $\mu (x)$ and $\sigma ^{2}(x)$ are the best current value (i.e. ${{\,\mathrm{arg\,min}\,}}_{x} f(x)$), predictive mean and predictive variance respectively; while $\Phi (\cdot )$, $\phi (\cdot )$ are the cumulative distribution function, probability density function respectively. $\kappa$ is a parameter that balances exploration against exploitation.

## Methods

### Implementation

We compared four conformational search algorithms: one systematic method as implemented by Confab [6] in Open Babel [33] (“Confab”); and three stochastic search methods: uniform random search (“Uniform”), plus two variants of Bayesian optimization algorithm (“BOA”) each with different acquisition functions: BOA with expected improvement (“EI”), and BOA with Gaussian process lower confidence bound (“LCB”). We used the Python package, GPyOpt, [34] for the Bayesian optimization algorithm variants and numpy [35] to generate random numbers between 0 and $2\pi$ for the uniform search. Pybel [36] was used to drive the torsion angles of the molecules for both uniform random search and BOA. We should note that the molecule’s bond lengths, bond angles, and ring systems remain unchanged throughout the search. Moreover, it is possible to sample torsion angles that generate steric clashes in the stochastic search, and it will return high energies.

All methods explored the same search space for each molecule, as determined by the set of freely-rotatable bonds in each. The search space of the algorithms was thus defined by a hypercube $[0,2\pi ]^{d}$, where *d* is the number of rotatable bonds in the molecule.

In order to compare all of the search algorithms fairly, we used the same number of iterations, *K* (i.e. number of conformers explored), for all of the stochastic search methods, i.e. uniform random search and BOA. We used $K=50$ for molecules with three or fewer rotatable bonds, and $K=100$ otherwise. Note that by the nature of the algorithm, BOA needs initial observations of the energy landscape in order to fit a Gaussian process. For each molecule, five observations were obtained by randomly sampling the conformational space at the beginning of the search. Hence only ($K-5$) conformers were evaluated after initial random sampling in BOA.

An energy cutoff of 500 kcal/mol was used in Confab, with up to one million conformers and a root mean square deviation clustering threshold of $0.05\; { \AA }$; all other Confab parameters were left as their default values. The RMSD cutoff of $0.05\; { \AA }$ was used so as to eliminate duplicate conformers with identical geometry to existing conformers. Note that only one compound (cochliodinol, a molecule with six rotatable bonds) would have generated more than one million conformers (1,327,104).

### Search duration

In order to understand how many energy evaluations are required to recover a better conformation or achieve high recovery rate, we investigated the effect of doubling the maximum number of energy evaluations, i.e. $K = 200$, in BOA search for the set of molecules with five rotatable bonds, and repeated the experiment four times for each molecule in the set.

### Torsion angle potentials and kernel

For each method, the torsion angles of all possible rotatable bonds in the molecule were used as model input variables and ranged from 0 to $2\pi$. Torsion angle preferences have previously been derived by Guba et al. [18] from commonly-occurring types of rotatable bonds observed in small molecule and protein-ligand X-ray crystal structures. We incorporated this prior knowledge into our Bayesian optimization algorithm using appropriately chosen kernels.

Specifically, we used a locally periodic kernel, i.e. a product of a periodic kernel and a squared exponential kernel. This allowed us to model torsional potentials with varying amplitudes as well as different local minima and maxima [37]. We derived the periodicity parameters for our kernels from the torsion potentials corresponding to 364 rotatable bond SMARTS patterns [18]. The periodicity for each pattern is given in Additional file 1. Note that when the list of patterns did not cover a specific type of rotatable bond, we assigned general values for the periodicity parameter based on the atomic hybridization of the two atoms in the rotatable bond, i.e. $sp^{2}-sp^{2}$, $sp^{2}-sp^{3}$, and $sp^{3}-sp^{3}$.

**Fig. 1:**
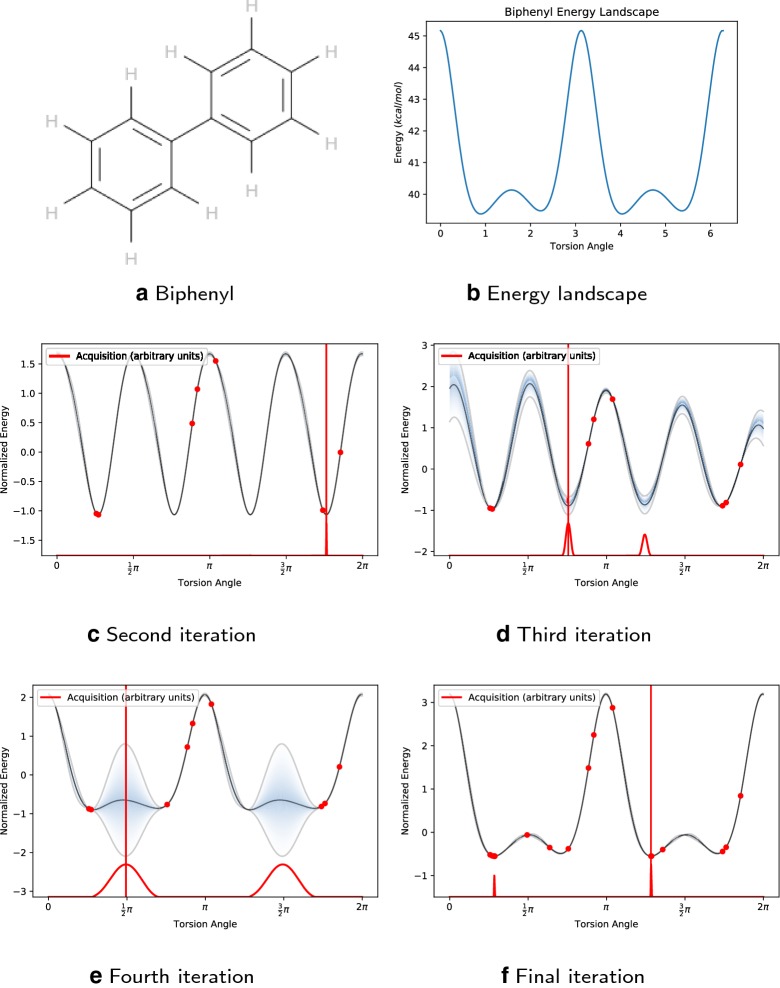
Example: **a** Biphenyl in 2D. **b** Simulated energy landscape under MMFF94. The blue line and the shaded blue region in **c**–**f** represent the mean function of the Gaussian process and express the uncertainty of the function respectively. The red points are the evaluated points. The red curve at the bottom of the graph shows the acquisition function, in particular expected improvement (EI) is used in this example. The red vertical line indicates the highest value of the EI, i.e. the point to be evaluated next. Note that the we normalized the output (energy) in Bayesian optimization algorithm, hence the normalized energy landscape is shown in **c**–**f**. **c** Second iteration, **d** third iteration, **e** fourth iteration, **f** final iteration

We illustrate the idea with a simple molecule, biphenyl (Fig. 1), which has one rotatable bond. Here we set a periodicity of two for the locally periodic kernel and chose expected improvement (EI) as the acquisition function; 15 iterations of BOA were used.

### Energy evaluation

The Merck Molecular Force Field, MMFF94 [38] was used to evaluate the energy of a given molecule as implemented in Open Babel 2.4.1 [33]. This is an approximation of the molecule’s actual energy landscape; ideally, we would use quantum chemical methods to compute the molecule’s energy as accurately as possible [39].

### Datasets

To benchmark the optimization performance of the search algorithms, we used the dataset assembled by Ebejer et al. [40], which consists of 708 distinct small molecules and includes ligands from the Astex diverse set [41]. We filtered this set for molecules with six or fewer rotatable bonds, giving a subset of 576 molecules, including four with no rotatable bonds. For each molecule, we generated conformers using Confab as implemented in Open Babel. The conformer with the lowest MMFF94 energy across all search methods was used as the reference conformation for that molecule. This criterion differs from that usually used to assess conformer generation algorithms, namely the X-ray crystallographic structure(s) of the small molecule. This is because the conformations observed in crystal structures are not necessarily the lowest energy conformation in the force field used for the search. Our task when evaluating search methods is to find the geometry that gives the lowest energy in the function we are exploring.

### Analysis

Three different measures were used to evaluate the performance of each search method, namely: (i) heavy atom root mean square deviation (RMSD); (ii) torsion fingerprint deviation (TFD) [42]; and (iii) the difference in MMFF94 energy ($\Delta E_{MMFF94}$).

*Root-mean-square deviation*

The atom-positional RMSD between the conformation of the reference molecule and the generated conformer was calculated as follows:

7 $$\begin{aligned} \text {RMSD} = \sqrt{\frac{1}{N_{atoms}}\sum _{i=1}^{N_{atoms}}(r_{i}-r_{i,ref})^{2}} \end{aligned}$$

where $N_{atoms}$ is the number of non-hydrogen atoms considered, $r_i$ is the position of atom *i* in the query conformer, and $r_{i,ref}$ is the corresponding position in the reference structure. The lowest MMFF94 energy conformation was used as the reference structure. Moreover, symmetry was taken into account when comparing molecules. For instance, both orientations of a benzene ring flipped by $180^{\circ }$ along its twofold symmetry axis would give an RMSD of $0 \;{ \AA }$. We used the RMSD calculation as implemented in Open Babel, Version 2.4.1.

*Torsion fingerprint deviation (TFD)*

Another way of comparing conformations is the torsion fingerprint deviation (TFD) and it is a non-superpositional method. The torsion angles of the non-terminal acyclic bonds and ring systems are extracted from two conformations and weighted according to their distance from the center of the molecule, and the difference is recorded. TFD values range from zero to one, with zero representing a perfect alignment of identical conformations. The topological weighting step ensures that changes of the torsional angle in the core of the molecule have more influence on TFD than changes toward the edges. Similarly, we used the lowest energy conformation as the reference conformation. We used the implementation of TFD in RDKit (2018.03.1) [10].

*Energy difference*

For each conformer, we computed the energy difference between the lowest energy conformation found by the search algorithm and that found by Confab. Negative values indicate a better conformation was found by the search than by Confab.

*Statistical tests*

The Wilcoxon signed-rank test was used to test whether the distributions of the lowest energy conformations found by each pair of search algorithms was statistically significantly different from one another, i.e. two-sided test. Here, the null hypothesis was that two related, paired samples (*x*, *y*), come from the same distribution. In particular, we compared three pairs of methods, namely (EI, Uniform), (LCB, Uniform), and (EI, LCB), and used a significance level of 5%.

## Results and discussion

We repeated each run of the stochastic search algorithms (Uniform, EI, and LCB) five times for each of 572 molecules. Four rigid molecules were ignored, and the results are summarized below. Note that due to occasional numerical instabilities, GPyOpt terminated early before reaching the maximum number of iterations requested. This was manifested by a non-positive definite kernel error. We separated out these molecules with “early stopping”, and are listed in Additional file 2. We also discuss possible solutions to address this issue “Gaussian process initialization in BOA” in section.

**Fig. 2:**
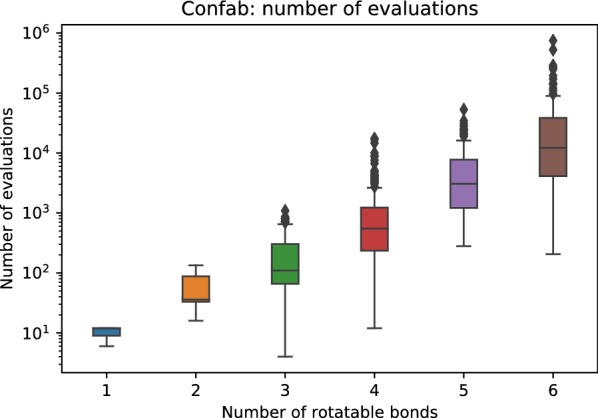
Distributions of the number of energy evaluations used by Confab versus the number of rotatable bonds

We first analyzed the number of conformers sampled by systematic search (Confab). Figure 2 shows that up to $10^{6}$ conformers were explored for molecules with six rotatable bonds. For molecules with four or more rotatable bonds, the median number of conformers generated was approximately $10^{3}$ to $10^{4}$. Cochliodinol has six rotatable bonds and had generated over one million conformers, with 750,402 conformers retained, and the lowest energy of 146.04 kcal/mol. Bayesian optimization algorithm, on the other hand, required only $10^{2}$ evaluations to obtain low energy conformations, and the best conformation out of five trials had an almost identical energy of 146.13 kcal/mol. This highlights the power of BOA, and we will show that BOA gives good performance in general despite using orders of magnitude fewer energy evaluations.

### Search performance

**Fig. 3:**
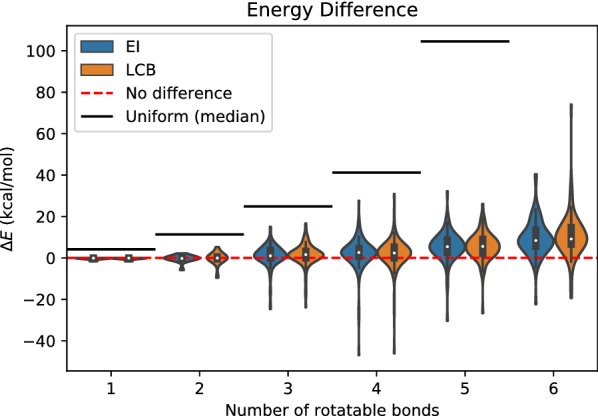
Energy difference versus number of rotatable bonds: BOA and uniform random search. The red dotted line indicates no energy difference between the lowest energy conformation found by the search algorithm and that found by Confab. The black line indicates the median energy difference of the uniform random search. Note that the median (213 kcal/mol) for molecules with six rotatable bonds is not shown in figure. The blue and orange groups show the result for BOA with EI, and BOA with LCB, respectively

Uniform random search performed the worst of all search methods. It gave higher median energy differences and larger ranges in energy difference than BOA search across all sets of rotatable bonds (Fig. 3). The distributions of the energy differences are very similar for BOA search, with both acquisition functions, EI and LCB. When constrained by a maximum number of energy evaluations, uniform random search suffers more in higher dimensions than BOA, and the median of the energy differences increases rapidly. On the other hand, the median of the energy differences in BOA search increases slowly and reaches approximately 9 kcal/mol for molecules with six rotatable bonds.

Confab was used to enumerate systematically all conformers for each molecule using the ‘torsion driving approach’. Confab iterates systematically through a set of allowed torsion angles for each rotatable bond in the molecule. Being a systematic search, Confab was thus expected to identify all the low energy conformations for each molecule. However, the best torsion angles may not be covered by the set of the torsion angles used in Confab. On the other hand, BOA samples torsion angles freely in the space and learns from the observed conformations, which enables it to recover conformations with lower energies using orders of magnitude fewer evaluations than Confab.

**Fig. 4:**
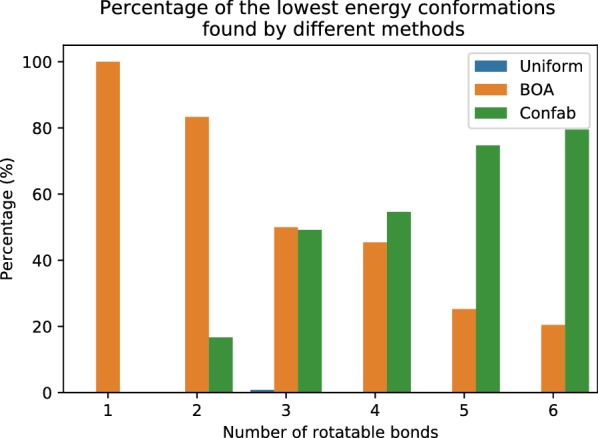
Frequency with which any method can find the lowest possible energy found by any of the search methods, from five independent trials. Note that Confab tends to use orders of magnitude more iterations than either BOA (EI and LCB) or uniform—the maximum number of iterations used for Confab is shown in Fig. 2, while a maximum of $10^{2}$ iterations was used for both BOA and uniform random search

**Fig. 5:**
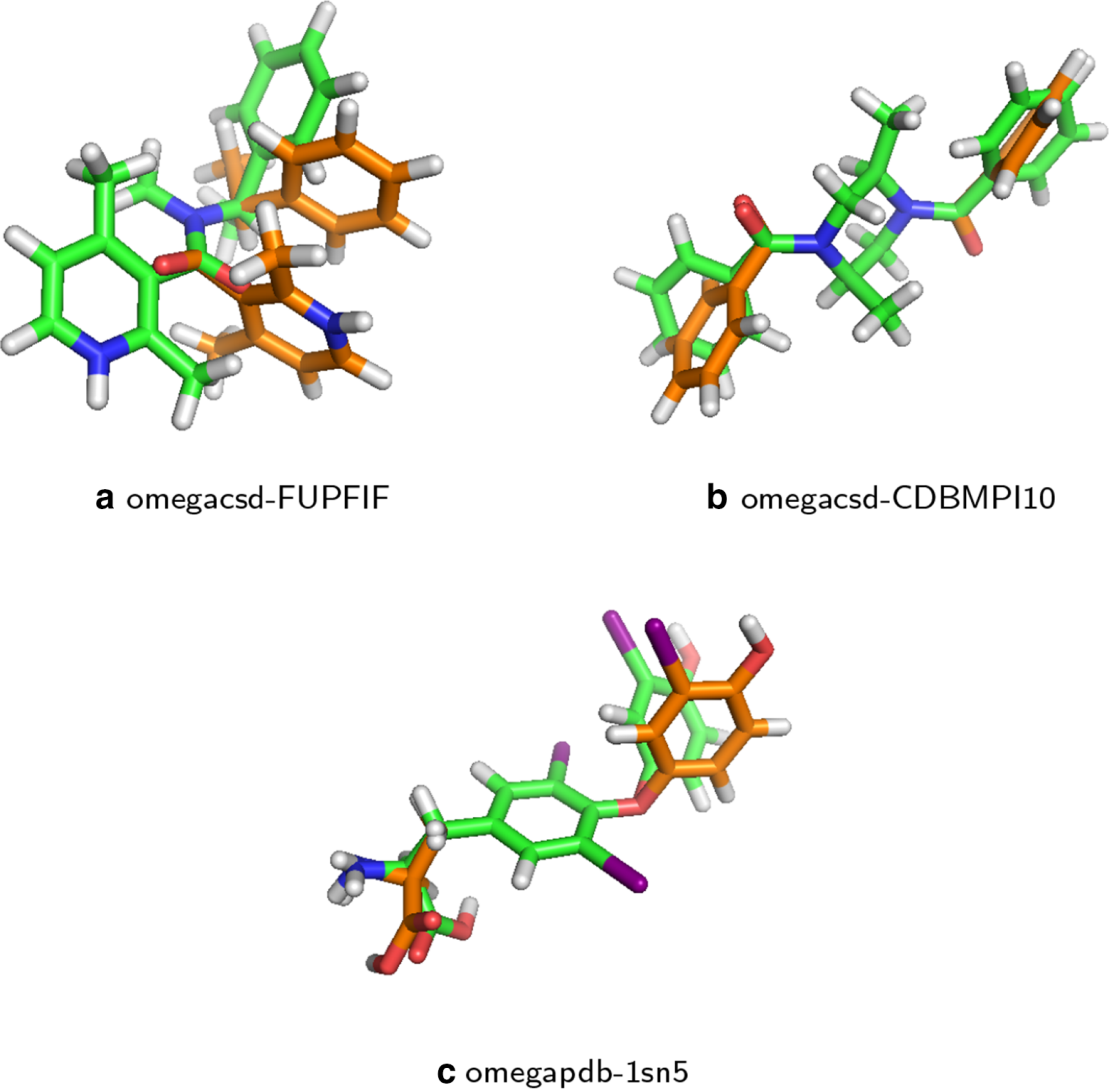
Examples where BOA found lower energies than Confab: **a** for omegacsd-FUPFIF, the lowest energy Confab found was 117 kcal/mol, while for BO, it was 70 kcal/mol; **b** for omegacsd-CDBMPI10, the lowest energy Confab found was 150 kcal/mol, while for BO: 118 kcal/mol; **c** for omegapdb-1sn5, the lowest energy Confab found was 131 kcal/mol, while for Bayesian optimization, it was: 99 kcal/mol. The lowest energy conformations found by Confab and BOA are shown in green and orange respectively. Figures are generated by PyMOL

We define the lowest energy conformer (LEC) for a given molecule as the lowest energy conformation found by any search method in our experiments. We computed the frequency that each method (Confab, BOA, and Uniform) was able to find each molecule’s LEC. Figure 4 shows that BOA recovers the most LECs for molecules with three or fewer rotatable bonds. This suggests that the geometries of the LECs deviate slightly from those with ideal torsion angles. It should be noted that these non-ideal conformers cannot be generated by Confab. Examples of molecules with conformations found by BOA that have significantly lower energies than those found by Confab are shown in Fig. 5. The proportion of LECs recovered by BOA decreased as the number of rotatable bonds increased. This is because BOA was limited to a maximum of 100 energy evaluations. Confab, on the other hand, had the opposite trend of that for BOA, as it was able to consume orders of magnitude more energy evaluations than BOA (Fig. 2).

**Fig. 6:**
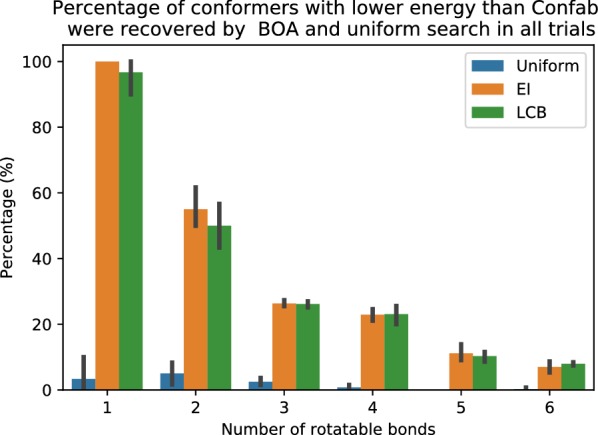
Champion rate: percentage of conformers with lower energy than Confab recovered by BOA with EI, BOA with LCB, and uniform random search. The error bars show the variation in the trials. It can be seen that both variants of BOA recover a lower energy conformer than Confab much more often than uniform random search, although the overall recovery rate drops as the number of rotatable bonds increases. A maximum of 100 iterations was used for molecules with four or more rotatable bonds and 50 iterations otherwise

We assessed the ‘champion rate’ of all search methods in each trial, i.e. the percentage of molecules that the search algorithm found better conformations than Confab in a single trial. Uniform random search had the lowest champion rate (see Fig. 6). We observed a similar rate in BOA search with both acquisition functions, EI and LCB. BOA search has a very high champion rate of 100% and 55% in molecules with one and two rotatable bonds, respectively. As expected, it decreases as the number of rotatable bonds increases. The champion rate is approximately 25% for molecules with three and four rotatable bonds, and 10% for molecules with five or more rotatable bonds. A key question here is how many samples are required to recover a better conformation and thus achieve a high recovery rate. We address the influence of the maximum number of evaluations “Search duration” in section.

#### Wilcoxon signed-rank test

**Table 1:** Energy difference: Wilcoxon signed-rank test on each method pair

Method-pair $N_{rot}$	1,2,3	4	5	6
Uniform-EI	${8.1\times 10^{-24}}$	${4.5\times 10^{-23}}$	${3.5\times 10^{-17}}$	${2.8\times 10^{-15}}$
Uniform-LCB	${4.5\times 10^{-24}}$	${4.5\times 10^{-23}}$	${3.7\times 10^{-17}}$	${2.6\times 10^{-15}}$
EI-LCB	${0.02}$	0.44	0.89	0.09

Molecules with three or fewer rotatable bonds ($N_{rot}: 1,2,3$) are grouped together due to small sample size. The *p*-values are rounded to 2 significant figures

The Wilcoxon signed-rank test of energy difference distributions (Table 1) showed that uniform random search is significantly different from BOA with EI and LCB (*p*-value $\ll$ 0.01) for all numbers of rotatable bonds. Note that the sample sizes of the sets of molecules with one and two rotatable bonds are small, and we combined these with molecules having three rotatable bonds for the statistical test. This gave more reliable test results. For the EI-LCB pair, we obtained a large *p*-value except for molecules with one to three rotatable bond (*p*-value of 0.02). Thus we found no evidence to reject the null hypothesis that the results for EI and LCB come from the same distribution.

Furthermore, we assessed the variation in energies found by BOA. In particular, we computed the maximum variation for each molecule, by extracting the lowest energy conformation found in each trial and computing the maximum difference in energy among these conformations. The results are summarized in Additional file 3. We observed a smaller variation in BOA than uniform search. The variation increases exponentially in uniform search while the variation increases gradually as the number of rotatable bonds increases in BOA search. The median reaches approximately 9 kcal/mol for molecules with six rotatable bonds in BOA search with EI and LCB. The range is larger in BOA with LCB than BOA with EI, except for molecules with five rotatable bonds.

#### RMSD and TFD

Both RMSD and TFD were used to measure the distance between reference conformer and that obtained by various search methods. The lowest energy conformation across all methods was used as the reference conformer, and two scenarios were considered. Case 1 considered the lowest energy conformation found by either BOA or uniform random search from all trials for each molecule, while Case 2 considered the lowest energy conformation found by Confab.

The conformers generated by uniform random search usually have higher RMSD and TFD values than those generated by BOA (see Additional file 3). The conformations found by BOA with both acquisition functions had similar distributions in RMSD and TFD values in Case 2, while EI and LCB slightly vary in Case 1.

Similarly, we grouped molecules with three or fewer rotatable bonds together. In addition, we combined molecules with five or more rotatable bonds together in Case (1) due to the small sample size in molecules with six rotatable bonds. Wilcoxon signed-rank tests for the RMSD and TFD distributions showed consistent results (see Additional file 4): the distribution of conformers generated by uniform random search is significantly different from that generated by BOA (*p* value $\ll$ 0.01). The conformers generated by BOA with both acquisition functions, EI and LCB, are not statistically different from each other.

### Search duration

**Fig. 7:**
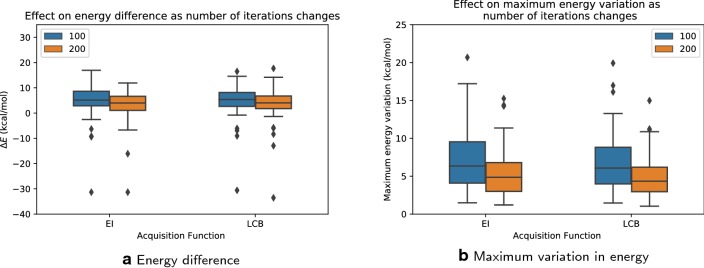
Effect of doubling the maximum number of energy evaluations: **a** energy difference; **b** maximum variation in energy. Note that only molecules with five rotatable bonds are tested. BOA with 100 iterations and 200 iterations are shown in blue and orange respectively. As expected, the energy difference and variation in energy decreases as number of energy valuations increases

We investigated the effect of doubling the maximum number of energy evaluations to 200 on the Bayesian optimization algorithm, for the set of molecules with five rotatable bonds. We found that the results were more robust and had smaller ranges of energetic differences than were found with 100 iterations. Figure 7 shows that the median of the energy difference distributions decreased by 1.1 kcal/mol for EI, and 1.3 kcal/mol for LCB. The maximum variation also decreased, by 1.5 kcal/mol for EI, and 1.7 kcal/mol for LCB. Thus, increasing the maximum number of iterations improves the likelihood of finding low-energy minima, and decreases the stochastic variance between multiple runs.

### Computational cost

**Fig. 8:**
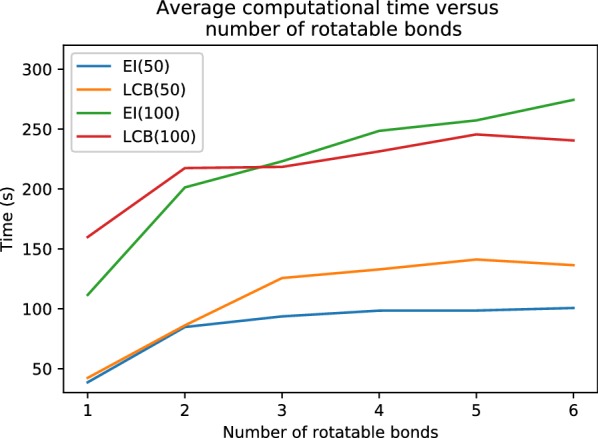
Average computational time for BOA with two difference acquisition function (EI or LCB) and different number of evaluations (50 or 100). Five molecules were randomly sampled for each rotor. The average computational time increases as number of rotatable bonds increase, but is primarily dominated by the number of conformers generated

Performance in terms of finding the lowest energy is improved by increasing the maximum number of energy evaluations. However, the computational cost also grows significantly (see Fig. 8). We should note that the computational complexity of the Gaussian process regression is $O(N^{3})$, where *N* is the number of evaluations. The run time analysis on BOA was performed on a desktop running Fedora 28 with an Intel Core i7-6700 operating at 3.40 GHz, and 32 GB of RAM. A single core was used for MMFF94 energy evaluation and driving the torsion angles. All cores were used in the GPyOpt operations. The time did not include the time to read input molecules or write the conformers to disk. It took about 7 ms to update the torsions and evaluate the energy 100 times in a molecule with six rotatable bonds. Hence, the computational time is dominated by the operations within GPyOpt. This is not surprising as we chose a relatively fast energy function, i.e. MMFF94. If the energy function was replaced by a more accurate but computationally more expensive method, such as a quantum mechanical method, we expect that the Bayesian optimization algorithm would become a more competitive search strategy than those used by other stochastic search methods.

In future work, we plan to explore two approaches to reduce computational cost: (a) using different surrogate models that have lower computational complexity [43, 44]; and (b) incorporating more accurate priors to improve the search speed.

### Alternative energy functions in BOA

The energy function explored by Bayesian optimization algorithm could be the result of a multi-step, “black box” protocol. For example, each energy value at a given point in the search space could be the result of a short MMFF94 optimization—not just a single-point energy calculation as used here (i.e. the common “fixed-rotor” approximation) [1]. In such a scenario, BOA would “learn” the torsion angles that minimize steric clashes in the molecule. This would not be comparable with the systematic Confab and uniform random search methods investigated here, because changes in bond lengths and bond angles introduced by the MMFF94 optimization would also change the energy landscape being optimized. Bond lengths and bond angles are never changed by Confab or torsion-driven uniform random search. This makes it hard to compare our current results with other stochastic search methods such as distance geometry, constrained distance geometry, and molecular mechanics energy minimization provided by tools such as RDKit. We intend to complete a comprehensive comparison with free, open-source, and commercial toolkits in the future, but note that at present among common tools, only the BOA strategy seeks to find the lowest energy conformer.

### Gaussian process initialization in BOA

Parameter initialization is important in GP regression. Poor initialization can easily lead to numerical instabilities, and several approaches can be used to address the issue: one would be to place priors on the parameters; alternatively, it may be possible to set boundary constraints on the parameters. The former approach would require more computational cost but would give a more robust estimation of the parameters.

## Conclusion

In this study, Bayesian optimization algorithm was used to find the lowest energy conformation for a set of 572 molecules with one to six rotatable bonds selected from the dataset assembled by Ebejer et al. [40]. Using this strategy, we have been able to incorporate our prior knowledge about torsion angle preferences extracted from crystal structures to accelerate the search for the lowest energy conformation.

We find that, by inherently sampling all possible dihedral angles, this approach often finds lower energy minima even below those generated by systematic enumeration, and with far fewer conformations. For small numbers of rotatable bonds (e.g., 1–3), BOA frequently finds lower energy conformations compared to systematic search in Confab. As the search space increase, BOA still finds lower energy geometries $\sim$20–40% of the time, despite many times fewer iterations.

More efficient methods for finding the lowest energy conformation of a small molecule will help accelerate the calculation of molecular properties and thus help to advance the fields of material design and drug discovery. Further studies are required to validate the search performance of BOA in higher dimensional space, and thus tackle more flexible molecules with more rotatable bonds.

## Additional file

**Additional file 1.** Library of rotatable bond SMARTS patterns.

**Additional file 2.** List of molecules that excluded from analysis.

**Additional file 3.** Figures for RMSD, TFD and maximum variation in energy.

**Additional file 4.** Tables of p-values for RMSD and TFD.

## Data Availability

All the data and code is available at https://figshare.com/articles/Conformers_Dataset/8120912 and GitHub https://github.com/lucianlschan/Conformer-Geometry respectively.

## Abbreviations

BOA: Bayesian optimization algorithm
GP: Gaussian process
EI: expected improvement
LCB: lower confidence bound
LEC: lowest energy conformer
RMSD: root mean square deviation
TFD: torsion fingerprint deviation
