# Supplementary material for: Bayesian optimization for conformer generation
Source: J Cheminform. 2019 May 21;11:32. doi: 10.1186/s13321-019-0354-7 (PMC6528340; doi:10.1186/s13321-019-0354-7)
Supplement: Supplementary file 1 — Additional file 1. Library of rotatable bond SMARTS patterns. [file 13321_2019_354_MOESM1_ESM.pdf]

	Rotatable bond SMARTS pattern	Parameter
0	[O:1]=[C:2]!@;-[O:3]~[CH0:4]	1
1	[O:1]=[C:2]([N])!@;-[O:3]~[C:4]	1
2	[O:1]=[C:2]!@;-[O:3]~[C:4]	1
3	[O:1]=[C:2]!@;-[O:3]~[!#1:4]	1
4	[$(C=O):1][O:2]!@;-[c:3]~[*:4]	2
5	[$(C=O):1][O:2]!@;-[CX3:3]~[*:4]	1
6	[$(C=O):1][O:2]!@;-[CH1:3][H:4]	1
7	[$(C=O):1][O:2]!@;-[CH2:3]~[C:4]	1
8	[H:1][CX4H1:2]!@;-[O:3][CX4:4]	1
9	[C:1][CH2:2]!@;-[O:3][CX4:4]	1
10	[*:1][CX4:2]!@;-[O:3][$([CX3](=[!O])):4]	1
11	[O:1][CX4:2]!@;-[O:3][CX4:4]	1
12	[*:1][CX4:2]!@;-[O:3][CX4:4]	1
13	[cH1:1][c:2]([cH1])!@;-[O:3][S:4]	2
14	[cH1:1][c:2]([cH0])!@;-[O:3][S:4]	2
15	[cH0:1][c:2]([cH0])!@;-[O:3][S:4]	2
16	[cH1:1][c:2]([cH1])!@;-[O:3][c:4]	1
17	[cH1:1][c:2]([cH0])!@;-[O:3][c:4]	1
18	[cH0:1][c:2]([cH0])!@;-[O:3][c:4]	2
19	[cH0:1][c:2]([cH0])!@;-[O:3][P:4]	2
20	[cH0:1][c:2]([cH0])!@;-[O:3][p:4]	2
21	[cH:1][c:2]([cH])!@;-[O:3][$(C([F])([F])[F]):4]	2
22	[cH0:1][c:2]([cH0])!@;-[O:3][CX4H0:4]	2
23	[a:1][c:2]([a])!@;-[O:3][CX4H0:4]	1
24	[cH1,n:1][c:2]!@;-[O:3][CRH1:4]	2
25	[cH1,n:1][c:2]!@;-[O:3][CH1:4]	2
26	[nX2H0:1][c:2]([cH0])!@;-[O:3][CX4H0:4]	1
27	[cH0:1][c:2]([nX2])!@;-[O:3][C:4]	1
28	[nX2:1][c:2]([nX2])!@;-[O:3][C:4]	2
29	[nX2:1][c:2]([nX3])!@;-[O:3][C:4]	1
30	[cH1:1][c:2]([nX3])!@;-[O:3][C:4]	1
31	[cH1:1][c:2]([nX2])!@;-[O:3][C:4]	1
32	[$([cH0]([CX3])):1][c:2]([cH1])!@;-[O:3][C:4]	1
33	[cH1:1][c:2](cO)!@;-[O:3][C:4]	1
34	[$(cO):1][c:2](cO)!@;-[O:3][C:4]	2
35	[cH0:1][c:2]([cH0])!@;-[O:3][C:4]	2
36	[cH0:1][c:2]([cH1])!@;-[O:3][C:4]	1
37	[cH1:1][c:2]([cH1])!@;-[O:3][C:4]	2
38	[a:1][c:2]!@;-[O:3][CX3H0:4]	2
39	[aH0:1][c:2]!@;-[OX2:3][!#1:4]	2
40	[!#1:1][CX4H0:2]!@;-[OX2:3][!#1:4]	1
41	[H:1][CX4H1:2]!@;-[OX2:3][!#1:4]	1

Continued on next page

	Rotatable bond SMARTS pattern	Parameter
42	[C:1][CX4H2:2]!@;-[OX2:3][c:4]	1
43	[c:1][CX4H2:2]!@;-[OX2:3][C:4]	1
44	[C:1][CX4H2:2]!@;-[OX2:3][C:4]	1
45	[c:1][CX4H2:2]!@;-[OX2:3][c:4]	1
46	[!#1:1][CX4H2:2]!@;-[OX2:3][c:4]	1
47	[!#1:1][CX4H2:2]!@;-[OX2:3][C:4]	1
48	[c:1][CX4H2:2]!@;-[OX2:3][!#1:4]	1
49	[C:1][CX4H2:2]!@;-[OX2:3][!#1:4]	1
50	[!#1:1][CX4H2:2]!@;-[OX2:3][!#1:4]	1
51	[!#1:1][CX4:2]!@;-[OX2:3][!#1:4]	2
52	[$([CX3]=O):1][NX3H0:2](C)!@;-[CX4H2:3][C:4]	2
53	[$([CX3]=O):1][NX3H1:2]!@;-[CX4H2:3][C:4]	1
54	[$(S(=O)(=O)):1][NX3H0:2]!@;-[CX4H2:3][!#1:4]	2
55	[$(S(=O)(=O)):1][NX3H1:2]!@;-[CX4H2:3][!#1:4]	1
56	[$(S(=O)(=O)):1][NX3H0:2]!@;-[CX4H1:3][H:4]	2
57	[$(S(=O)(=O)):1][NX3H1:2]!@;-[CX4H1:3][H:4]	1
58	[$(S(=O)(=O)):1][NH1:2]!@;-[c:3][nX2:4]	2
59	[$(S(=O)(=O)):1][NH0:2]!@;-[c:3]([cH1])[cH1:4]	2
60	[$(S(=O)(=O)):1][NH1:2]!@;-[c:3]([cH1])[cH1:4]	1
61	[$(S(=O)(=O)):1][NH0:2]!@;-[c:3]([cH1])[cH0:4]	2
62	[$(S(=O)(=O)):1][NH1:2]!@;-[c:3]([cH1])[cH0:4]	1
63	[$(S(=O)(=O)):1][NH0:2]!@;-[c:3]([cH0])[cH0:4]	2
64	[$(S(=O)(=O)):1][NH1:2]!@;-[c:3]([cH0])[cH0:4]	2
65	[$(S(=O)(=O)):1][N:2]!@;-[c:3][a:4]	1
66	[O-:1][N+:2](=O)!@;-[c:3]([cH,nX2H0])[cH,nX2H0:4]	2
67	[O-:1][N+:2](=O)!@;-[c:3]([cH0])[cH,nX2H0:4]	2
68	[O-:1][N+:2](=O)!@;-[c:3]([cH0])[cH0:4]	2
69	[O-:1][N+:2](=O)!@;-[c:3][a:4]	2
70	[cH0:1][c:2]([cH0])!@;-[NX3H1:3][C,c:4](~[N,n]...	2
71	[cH0:1][c:2]([cH1])!@;-[NX3H1:3][C,c:4](~[N,n]...	1
72	[cH0:1][c:2]([nX2H0])!@;-[NX3H1:3][C,c:4](~[N,...	2
73	[cH1:1][c:2]([cH1])!@;-[NX3H1:3][C,c:4](~[N,n]...	2
74	[nX2H0:1][c:2]([nX2H0])!@;-[NX3H1:3][C,c:4](~[...	2
75	[nX2H0:1][c:2]([nX3H1])!@;-[NX3H1:3][C,c:4](~[...	2
76	[a:1][a:2]!@;-[NH1:3][C,c:4](~[N,n])(~[N,n])	2
77	[C:1][NH:2]!@;-[C:3](=[NH2:4])[NH2]	2
78	[NH2][C:1](=[NH2])[NH:2]!@;-[CH2:3][C:4]	1
79	[a:1][c:2]!@;-[NX2:3]=[$(C([NX3])n):4]	2
80	[nX2:1][c:2]!@;-[NX2:3]=[$(C([NX3])N):4]	2
81	[cH0:1][c:2]!@;-[NX2:3]=[$(C([NX3])N):4]	2
82	[cH1:1][c:2]!@;-[NX2:3]=[$(C([NX3])N):4]	2
83	[O:1]=[C:2]([NH1])!@;-[NX3H1:3](C=O)[H:4]	1

Continued on next page

	Rotatable bond SMARTS pattern	Parameter
84	[O:1]=[C:2]!@;-[NX3H1:3](C=O)[H:4]	2
85	[O:1]=[C:2]!@;-[NX3:3](C=O)[*:4]	2
86	[$(C=O):1][NX3H1:2]!@;-[CX3:3]=[NX2:4]	2
87	[$(C=O):1][NX3H0:2]!@;-[CX3:3]=[*H0:4]	2
88	[$(C=O):1][NX3H0:2]!@;-[CX3:3]=[*H1:4]	1
89	[$(C=O):1][NX3H1:2]!@;-[CX3:3]=[*H2:4]	2
90	[$(C=O):1][NX3H1:2]!@;-[CX3:3]=[*H1:4]	1
91	[$(C=O):1][NX3H1:2]!@;-[CX3:3]=[*H0:4]	2
92	[$(C=O):1][NX3H0:2]!@;-[CX3H1:3]=[*:4]	1
93	[$(C=O):1][NX3H1:2]!@;-[CX3H1:3]=[*:4]	1
94	[$([C](=O)):1][NX3H0:2]!@;-[CX4H2:3][$([c]([cH...	2
95	[$(C=O):1][NX3H1:2]!@;-[CX4H2:3][$([c]([cH,nX2...	2
96	[$(C=O):1][NX3H0:2]!@;-[CX4H2:3][!#1:4]	2
97	[$(C=O):1][NX3H1:2]!@;-[CX4H2:3][!#1:4]	1
98	[$(C=O):1][NX3H0:2]!@;-[CX4H1:3][H:4]	2
99	[$(C=O):1][NX3H1:2]!@;-[CX4H1:3][H:4]	1
100	[$(C=O):1][NX3H0:2]!@;-[CX4H0:3][C:4]	1
101	[$(C=O):1][NX3H1:2]!@;-[CX4H0:3][C:4]	1
102	[$(C=O):1][NX3:2]!@;-[!#1:3][!#1:4]	1
103	[$([C](=O)([$([NX3H1]),$([NX3H2])])[NX3H1]):1]...	1
104	[$([C](=O)([$([NX3H1]),$([NX3H2])])[NX3H1]):1]...	1
105	[$([C](=O)):1][NX3H1:2]!@;-[$([a]([nH0,o])):3]...	1
106	[$([C](=O)([$([NX3H1]),$([NX3H2])])[NX3H1]):1]...	2
107	[$([C](=O)):1][NX3H1:2]!@;-[c:3]([cH])[nX2H0:4]	1
108	[$(C=O):1][NX3H0:2]!@;-[c:3]([s,o])[n:4]	1
109	[$(C=O):1][NX3H1:2]!@;-[c:3]([s,o])[n:4]	1
110	[$([C](=O)):1][NX3:2]!@;-[a:3](s)[a:4]	1
111	[$(C=O):1][NX3:2]!@;-[a:3][nH:4]	1
112	[$(C=O):1][NX3H1:2]!@;-[c:3]([cH0]Cl)[cH:4]	1
113	[$(C=O):1][NX3H1:2]!@;-[c:3]([cH0]F)[cH:4]	1
114	[$(C=O):1][NX3:2]!@;-[$([a]([cH1])):3][$([aH0]...	1
115	[$(C=O):1][NX3:2]!@;-[a:3][aH0:4]	1
116	[$(C=O):1][NX3H1:2]!@;-[c:3]([cH1])[cH1:4]	2
117	[$(C=O):1][NX3H0:2]!@;-[c:3]([cH1])[cH1:4]	2
118	[$(C=O):1][NX3H0:2]!@;-[c:3]([cH0])[cH:4]	2
119	[$(C=O):1][NX3H1:2]!@;-[c:3]([cH0])[cH:4]	1
120	[$(C=O):1][NX3H0:2]!@;-[c:3]([cH0])[cH0:4]	2
121	[$(C=O):1][NX3H1:2]!@;-[c:3]([cH0])[cH0:4]	2
122	[O,S:1]=[C:2]([$([NX3H1]),$([NX3H2])])!@;-[$([...	2
123	[O:1]=[C:2]!@;-[$([NX3](c([nH1])n)):3][H:4]	1
124	[O:1]=[C:2](c)!@;-[$([NX3](c([nX2H0])([nX2H0])...	2
125	[O:1]=[CX3:2]([$([NX3H1]C)])!@;-[NX3H1:3][!#1:4]	1

Continued on next page

	Rotatable bond SMARTS pattern	Parameter
126	[O:1]=[C:2]([!$([NH1])])!@;-[NX3H1:3]([H:4])[$...	1
127	[O,S:1]=[C:2]([$([NX3H1]),$([NX3H2])])!@;-[$([...	2
128	[O:1]=[C:2]!@;-[NX3H0:3]([a:4])[A]	2
129	[O:1]=[CX3:2](a)!@;-[NX3H0:3][!#1:4]	2
130	[O:1]=[CX3:2]!@;-[NX3H0:3][!#1:4]	2
131	[O:1]=[CX3:2]!@;-[NX3H1:3][!#1:4]	1
132	[CH0:1][NX3:2]([CH0])!@;-[c:3][a:4]	2
133	[CH0:1][NX3:2]([CH1])!@;-[c:3][a:4]	2
134	[cH1,nX2H0:1][c:2]([cH1,nX2H0])!@;-[NX3&r:3][*:4]	2
135	[a:1][c:2]!@;-[NX3H1:3][$([CX4&r]([C;r])([C;r]...	2
136	[cH1:1][c:2]([cH1])!@;-[NX3:3][CX4:4]	2
137	[cH0:1][c:2]([cH,nX2H0])!@;-[NX3H1:3][CX4:4]	1
138	[cH0:1][c:2]([cH,nX2H0])!@;-[NX3H0:3][CX4:4]	1
139	[cH0:1][c:2]([cH0])!@;-[NX3:3][CX4:4]	1
140	[c:1][c:2](c)!@;-[NX3:3][CX4:4]	1
141	[cH1:1][c:2]([cH1])!@;-[NX3:3][a:4]	2
142	[cH1:1][c:2]([cH0])!@;-[NX3:3][a:4]	1
143	[cH0:1][c:2]([cH0])!@;-[NX3:3][a:4]	2
144	[cH0:1][a:2]!@;-[NX3H0:3][$([CX3]=O):4]	2
145	[cH0:1][a:2]!@;-[NX3H1:3][$([CX3]=O):4]	1
146	[nX2H0:1][a:2]([nX2H0])!@;-[NX3H0:3][$([CX3]=O...	1
147	[nX2H0:1][$(a([!nX2H0])([nX2H0])!@;-[NX3H1]):2...	1
148	[nX2H0:1][a:2]!@;-[NX3H1:3][$([CX3]=O):4]	2
149	[a:1][a:2]!@;-[NX3H1:3][$([CX3]=O):4]	2
150	[a:1][a:2]!@;-[NX3:3][CX4H0:4]	1
151	[a:1][a:2]!@;-[NX3:3][!#1:4]	2
152	[O:1]=[CX3:2]!@;-[nX3:3]([aH0:4])([aH0])	2
153	[O:1]=[CX3:2]!@;-[nX3:3][aH1:4]	2
154	[a:1][CX3:2](=S)!@;-[NX3:3][a:4]	1
155	[!#1:1][CX3:2](=S)!@;-[NX3H0:3][!#1:4]	2
156	[!#1:1][CX3:2](=S)!@;-[NX3H1:3][!#1:4]	2
157	[!#1:1][CH2:2]!@;-[n:3][cH0:4]	2
158	[!#1:1][CH2:2]!@;-[n:3][a:4]	2
159	[cH0:1][n:2]!@;-[CX3H0:3]~[$([n,N](-a)):4]	1
160	[CX4:1][CX4H2:2]!@;-[NX3:3][CX4:4]	1
161	[C:1][CX4H2:2]!@;-[NX3:3][C:4]	1
162	[C:1][CX4:2]!@;-[NX3:3][C:4]	1
163	[!#1:1][CX4H2:2]!@;-[NX3H1:3][!#1:4]	1
164	[!#1:1][CX4H2:2]!@;-[NX3:3][!#1:4]	1
165	[!#1:1][CX4H1:2]!@;-[NX3:3][!#1:4]	1
166	[!#1:1][CX4:2]!@;-[NX3:3][!#1:4]	1
167	[!#1:1][$(S(=O)=O):2]!@;-[nX3:3]([aH1])[aH1:4]	2

Continued on next page

	Rotatable bond SMARTS pattern	Parameter
168	[!#1:1][$(S(=O)=O):2]!@;-[nX3:3][aH0:4]	2
169	[c:1][S:2](=O)(=O)!@;-[NX2H0-:3]-[*:4]	2
170	[*:1][$(S(=O)=O):2]!@;-[NX3H0&r:3][*:4]	2
171	[C:1][$(S(=O)=O):2]!@;-[NX3H1:3][c:4]	2
172	[C:1][$(S(=O)=O):2]!@;-[NX3H0:3][c:4]	2
173	[c:1][$(S(=O)=O):2]!@;-[NX3H1:3][C:4]	2
174	[c:1][$(S(=O)=O):2]!@;-[NX3H0:3][C:4]	2
175	[c:1][$(S(=O)=O):2]!@;-[NX3H1:3][c:4]	2
176	[c:1][$(S(=O)=O):2]!@;-[NX3H0:3][c:4]	2
177	[C:1][$(S(=O)=O):2]!@;-[NX3H1:3][C:4]	2
178	[C:1][$(S(=O)=O):2]!@;-[NX3H0:3][C:4]	2
179	[*:1][$(S(=O)=O):2]!@;-[NX3H1:3][*:4]	2
180	[*:1][$(S(=O)=O):2]!@;-[NX3H0:3][*:4]	2
181	[!#1:1][CX3:2]!@;-[SX2:3][!#1:4]	2
182	[!#1:1][CX4:2]!@;-[SX2:3][!#1:4]	2
183	[!#1:1][CX3:2]!@;-[SX3:3][!#1:4]	2
184	[!#1:1][CX4:2]!@;-[SX3:3][!#1:4]	1
185	[!#1:1][CX3:2]!@;-[SX4:3][!#1:4]	2
186	[H:1][CX4H1:2]!@;-[SX4:3][!#1:4]	1
187	[!#1:1][CX4:2]!@;-[SX4:3][!#1:4]	1
188	[aH1:1][c:2]([aH1])!@;-[SX2:3][!#1:4]	1
189	[aH1:1][c:2]([aH0])!@;-[SX2:3][*R:4]	1
190	[aH1:1][c:2]([aH0])!@;-[SX2:3][!#1:4]	1
191	[aH0:1][c:2]([aH0])!@;-[SX2:3][!#1:4]	1
192	[!#1:1][c:2]!@;-[SX2:3][!#1:4]	2
193	[!#1:1][c:2]!@;-[SX3:3][!#1:4]	2
194	[aH1:1][c:2]([aH1])!@;-[SX4:3][!#1:4]	2
195	[aH0:1][c:2]([aH1])!@;-[SX4:3][!#1:4]	1
196	[aH0:1][c:2]([aH0])!@;-[SX4:3][!#1:4]	2
197	[O:1]=[CX3:2]([NH1])!@;-[CH2:3][CX3:4]=O	1
198	[O:1]=[CX3:2]([NH1])!@;-[CH2:3][C:4]	1
199	[$([CX3]([C])([H])):1]=[CX3:2]([C])!@;-[CH2:3]...	1
200	[$([CX3]([C])([H])):1]=[CX3:2]([H])!@;-[CH1:3]...	1
201	[$([CX3]([C])([H])):1]=[CX3:2]([H])!@;-[CH2:3]...	1
202	[N:1][C:2](=O)!@;-[CX4H2:3][CX4H2:4]	1
203	N[C:2](=[O:1])!@;-[CH2:3][N:4]	2
204	[O:1]=[C:2]([O-])!@;-[CX4H1:3][H:4]	1
205	[CX3H2:1]=[CX3:2]!@;-[CX3:3]=[C:4]	1
206	[CX3:1]=[CX3:2]!@;-[CH2:3][OX2:4]	1
207	[CX3:1]=[CX3:2]!@;-[CH1:3](C)[C:4]	1
208	[CX3:1]=[CX3:2]!@;-[CH2:3][C:4]	1
209	[CX3:1]=[CX3:2]!@;-[CH2:3][c:4]	1

Continued on next page

	Rotatable bond SMARTS pattern	Parameter
210	[CX3:1]=[CX3:2]!@;-[CH2:3][!#1:4]	1
211	[O:1]=[CX3:2](O)!@;-[CX3:3]([$([NH1,NH2,CH2])]...	2
212	[O:1]=[CX3:2]!@;-[CX3:3]=[O:4]	1
213	[CX3R:1]=[CX3R:2]!@;-[CX3:3]=[CX3:4]	2
214	[CX3H0:1]=[CX3H0:2]!@;-[CX3:3]=[CX3H0:4]	1
215	[CX3H0:1]=[CX3H0:2]!@;-[CX3H0:3]=[CX3:4]	2
216	[CX3H0:1]=[CX3:2]!@;-[CX3H0:3]=[CX3:4]	1
217	[CX3H0:1]=[CX3H0:2]!@;-[CX3:3]=[CX3:4]	1
218	[CX3:1]=[CX3:2]!@;-[CX3:3]=[CX3:4]	1
219	[*^2:1]~[C^2:2]([!H])!@;-[C^2:3]~[*^2:4]	2
220	[*^2:1]~[C^2:2]!@;-[C^2:3]~[*^2:4]	2
221	[O:1]=[CX3:2]!@;-[CX4&r3:3]!@[!#1:4]	2
222	[O:1]=[CX3:2]!@;-[CX4H1&r3:3][H:4]	2
223	[OX2:1][CX4H2:2]!@;-[CX4H2:3][N&r:4]	1
224	[OX2:1][CX4H2:2]!@;-[CX4H2:3][N:4]	1
225	[OX2:1][CX4:2]!@;-[CX4:3][N:4]	1
226	[OX2:1][CX4H2:2]!@;-[CX4H2:3][OX2:4]	1
227	[OX2:1][CX4:2]!@;-[CX4:3][OX2:4]	1
228	[!#1:1][CX4&r:2]!@;-[CX4&r:3][!#1:4]	1
229	[!#1:1][CX4H2:2]!@;-[CX4H2:3][!#1:4]	1
230	[!#1:1][CX4:2]!@;-[CX4:3][!#1:4]	1
231	[OX2:1][CX4H2:2]!@;-[CX3:3]([$([NX3H1,NX3H2])]...	2
232	[OH1:1][CX4:2]!@;-[CX3:3]=[O:4]	2
233	[NH1:1][CX4:2]!@;-[CX3:3]=[O:4]	2
234	[O:1][CX4:2]!@;-[CX3:3]=[O:4]	2
235	[N:1][CX4:2]!@;-[CX3:3]=[O:4]	2
236	[C:1][CX4H2:2]!@;-[CX3:3]=[O:4]	1
237	[c:1][CX4H2:2]!@;-[CX3:3]=[O:4]	1
238	[!#1:1][CX4H2:2]!@;-[CX3:3]=[O:4]	2
239	[c:1][CX4:2]!@;-[CX3:3]=[O:4]	1
240	[C:1][CX4:2]!@;-[CX3:3]=[O:4]	1
241	[!#1:1][CX4:2]!@;-[CX3:3]=[O:4]	1
242	[c:1][CX4:2]!@;-[CX3:3][C:4]	1
243	[C:1][CX4:2]!@;-[CX3:3][c:4]	1
244	[c:1][CX4:2]!@;-[CX3:3][c:4]	2
245	[C:1][CX4:2]!@;-[CX3:3][C:4]	1
246	[!#1:1][CX4:2]!@;-[CX3H0:3][!#1:4]	1
247	[H:1][CX4H1:2]!@;-[CX3:3][!#1:4]	2
248	[!#1:1][CX4H2:2]!@;-[CX3:3][!#1:4]	1
249	[$([cH0]([$([NX3H2]),$([NX3H1])])):1][a:2]!@;-...	2
250	[nH0:1][c&r6:2]([nH0])!@;-[c&r6:3]([nH0])[nH0:4]	2
251	[nH0&r6:1][c&r6:2]([nH0&r6])!@;-[c&r6:3]([cH1&...	2

Continued on next page

	Rotatable bond SMARTS pattern	Parameter
252	[nH0&r6:1][c&r6:2]([nH0&r6])!@;-[c&r6:3]([cH1&...	2
253	[c:1][c:2]!@;-[c:3][$(c!@c):4]	2
254	[cH0:1][c:2]([cH0])!@;-[c:3]([cH0:4])[cH0]	2
255	[cH0:1][c:2]([cH0])!@;-[c:3]([cH0:4])[cH1]	2
256	[cH0:1][c:2]([cH1])!@;-[c:3]([cH0:4])[cH1]	2
257	[cH0:1][c:2]([cH0])!@;-[c:3]([cH1:4])[cH1]	2
258	[cH0:1][c:2]([cH1])!@;-[c:3]([cH1:4])[cH1]	1
259	[cH1:1][c:2]([cH1])!@;-[c:3]([cH1:4])[cH1]	2
260	[nX2H0:1][c:2]!@;-[c:3][nX3H1:4]	2
261	[nX2H0:1][c:2]([!nX2H0])!@;-[c:3]([!nX2H0])[nX...	1
262	[nX2H0:1][$(c([nX2H0])(a(a)(a))!@;-c[nX2H0]):2...	1
263	[nX2H0:1][$([c&r6](c[OH])):2]!@;-[$([c&r6](c[O...	1
264	[c:1][c:2]!@;-[c:3][s,o,nX3H1:4]	1
265	[cH0:1][c:2]([cH0])!@;-[c:3][nX2H0:4]	2
266	[cH0:1][c:2]!@;-[c:3]([cH0])[nX2H0:4]	2
267	[c:1][c:2]!@;-[c:3]([cH0])[nX2H0:4]	1
268	[c:1][c:2]([cH0])!@;-[c:3][nX2H0:4]	2
269	[cH1:1][c:2]!@;-[c:3]([cH1])[nX2H0:4]	2
270	[c:1][c&r5:2]!@;-[c&r5:3][c:4]	2
271	[c:1][c&r6:2]!@;-[c&r5:3][c:4]	1
272	[c:1][c&r6:2]!@;-[c&r6:3][cH0:4]	2
273	[c:1][c&r6:2]!@;-[c&r6:3][c:4]	1
274	[c:1][c:2]!@;-[c:3][c:4]	1
275	[nX2&r6:1][cH0&r6:2]([cH1&r6])!@;-[CX4H2:3][O!...	1
276	[cH0:1][c:2]!@;-[CX4H0:3][a:4]	1
277	[cH0:1][c:2]!@;-[CX4H0:3][N,O,S:4]	1
278	[cH0:1][c:2]!@;-[CX4H0:3][CX3:4]	1
279	[cH0:1][c:2]!@;-[CX4H0:3][CX4:4]	1
280	[cH0:1][c:2]!@;-[CX4H0:3][*:4]	1
281	[cH0:1][c:2]!@;-[CX4H2:3][a:4]	2
282	[cH0:1][c:2]!@;-[CX4H2:3][CX3:4]	2
283	[cH1:1][c:2]([cH1])!@;-[CX4H2:3][$([CX4H1]C(=O...	2
284	[cH1:1][c:2]([cH1])!@;-[CX4H2:3][CX4:4]	2
285	[cH0:1][c:2]!@;-[CX4H2:3][CX4:4]	2
286	[cH0:1][c:2]!@;-[CX4H2:3][N,O,S:4]	2
287	[cH0:1][c:2]!@;-[CX4H2:3][!#1:4]	2
288	[cH0:1][c:2]!@;-[CX4H1:3][a:4]	2
289	[cH0:1][c:2]!@;-[CX4H1:3][CX4:4]	2
290	[cH0:1][c:2]!@;-[CX4H1:3][CX3:4]	1
291	[cH0:1][c:2]!@;-[CX4H1:3][N,O,S:4]	1
292	[cH0:1][c:2]!@;-[CX4H1:3][H:4]	2
293	[a:1][c:2]!@;-[CX4H0:3][a:4]	2

Continued on next page

	Rotatable bond SMARTS pattern	Parameter
294	[a:1][c:2]!@;-[CX4H0:3][CX3:4]	1
295	[a:1][c:2]!@;-[CX4H0:3][CX4:4]	1
296	[a:1][c:2]!@;-[CX4H0:3][N,O:4]	2
297	[a:1][c:2]!@;-[CX4H2:3][a:4]	2
298	[a:1][c:2]!@;-[CX4H2:3][CX3:4]	2
299	[n,o,s:1][c:2]!@;-[CX4H2:3][CX4:4]	1
300	[a:1][c:2]!@;-[CX4H2:3][!#1:4]	2
301	[a:1][c:2]!@;-[CX4H1:3][N,O:4]	1
302	[a:1][c:2]!@;-[CX4H1:3][a:4]	2
303	[a:1][c:2]!@;-[CX4H1:3][H:4]	2
304	[nX2H0:1][c:2]!@;-[C:3](=[N:4])(-[NH1,NH2])	2
305	[a:1][c:2]!@;-[C:3](=[$([N][CX4]):4])([$(N[CX4...	2
306	[a:1][c:2]!@;-[C:3](=[$([NH0][CX4]):4])(-N)	2
307	[a:1][c:2]!@;-[C:3](=[$([N][!#1]):4])([$(N(C)[...	2
308	[a:1][c:2]!@;-[C:3](=[$([N][!#1]):4])([$(N(C)~...	2
309	[a:1][c:2]!@;-[C:3](=[N:4])(-N)	2
310	[O:1]=[C:2]([O-])!@;-[c:3][$(aC(=O)(O)):4]	2
311	[O:1]=[C:2]([O-])!@;-[c:3][nX3H1:4]	2
312	[O:1]=[C:2]([O-])!@;-[c:3][nX2H0:4]	2
313	[O:1]=[C:2]([O-])!@;-[c:3]([cH0])[cH0:4]	1
314	[O:1]=[C:2]([O-])!@;-[c:3]([cH1])[$([cH0][NH1,...	2
315	[O:1]=[C:2]([O-])!@;-[c:3]([cH1])[cH0:4]	2
316	[O:1]=[C:2]([O-])!@;-[c:3]([cH1])[cH1:4]	2
317	[O:1]=[C:2]([O-])!@;-[c:3][a:4]	2
318	[$([c]([NH1,NH2])):1][c:2]!@;-[CX3:3]([!O])=[O:4]	1
319	[$(a[OH1]):1][a:2]!@;-[CX3:3]([NX3H0,CX4H0,c])...	1
320	[$(a[NH1,NH2]):1][a:2]!@;-[CX3:3]([NX3H0,CX4H0...	1
321	[cH0:1][c:2]([cH1])!@;-[CX3:3](c)=[O:4]	1
322	[cH1:1][c:2]([cH1])!@;-[CX3:3](c)=[O:4]	2
323	[a:1][a:2]!@;-[CX3:3](a)=[O:4]	1
324	[$([cH0](=O)):1][c:2]([cH1])!@;-[CX3:3]([NX3H1...	1
325	[nH0&r6:1][c&r6:2]([cH1&r6])!@;-[C:3]([NH1,NH2...	1
326	[s:1][c:2]!@;-[C:3]([NH1])=[O:4]	2
327	[$([cH0]Cl):1][c:2]([cH1])!@;-[CX3:3]([NX3H1])...	2
328	[$([cH0]F):1][c:2]([cH1])!@;-[CX3:3]([NX3H1])=...	1
329	[$([cH0][OH0]):1][c:2]([cH1])!@;-[C:3](=O)[NH1:4]	1
330	[$([cH0][OH1]):1][c:2]([cH1])!@;-[C:3](=O)[NH1:4]	2
331	[cH0:1][c:2]([cH1])!@;-[CX3:3]([NX3H1])=[O:4]	1
332	[cH0:1][c:2]([cH1])!@;-[CX3:3]([NX3H0])=[O:4]	2
333	[cH1:1][c:2]([cH1])!@;-[C:3]([NH1,NH2])=[O:4]	2
334	[a:1][c:2]!@;-[C:3]([NH0])=[O:4]	1
335	[a:1][c:2]!@;-[C:3]([NH1,NH2])=[O:4]	2

Continued on next page

	Rotatable bond SMARTS pattern	Parameter
336	[s:1][c:2]([aX2,cH1])!@;-[CX3:3](O)=[O:4]	2
337	[s:1][c:2]([aX2,cH1])!@;-[CX3:3]=[O:4]	1
338	[$([cH0](F)):1][c:2]([cH1])!@;-[CX3:3]([O,N])=...	2
339	[$([cH0]F):1][c:2]([cH1])!@;-[CX3:3]=[O:4]	1
340	[$([cH0](Cl)):1][c:2]([cH1])!@;-[CX3:3]([CX3H]...	1
341	[$([cH0](Cl)):1][c:2]([cH1])!@;-[CX3H:3]=[O:4]	1
342	[$([cH0](Cl)):1][c:2]([cH1])!@;-[CX3:3](O)=[O:4]	2
343	[nX3H1:1][a:2]!@;-[CX3:3]=[O:4]	2
344	[nX2H0:1][c:2]([cH1])!@;-[CX3H1:3]=[O:4]	1
345	[nX2H0&r6:1][c&r6:2]([c&r6])!@;-[CX3:3]([!O])=...	1
346	[nX2H0:1][a:2]([nX2H0])!@;-[CX3:3]=[O:4]	2
347	[$([cH0]!@;-[*^2]):1][c:2]([cH1])!@[CX3:3]=[O:4]	2
348	[cH0:1][c:2]([cH1])!@;-[CX3:3]=[O:4]	2
349	[cH0:1][c:2]([cH0])!@;-[CX3:3]=[O:4]	1
350	[cH1:1][c:2]([cH1])!@;-[CX3:3]([CX3H0])=[O:4]	2
351	[cH1:1][c:2]([cH1])!@;-[CX3:3]=[O:4]	2
352	[a:1][a:2]!@;-[CX3:3]=[O:4]	2
353	[cH1:1][c:2]([nX2])!@;-[CX3:3]=[NX3:4]	2
354	[cH1:1][c:2]([nX3H1])!@;-[CX3:3]=[NX2:4]	1
355	[cH1:1][c:2]([nX2])!@;-[CX3:3]=[NX2:4]	2
356	[cH1:1][c:2]([$([cH0][OH1])])!@;-[CX3:3]=[NX2:4]	1
357	[cH1:1][c:2]([cH0])!@;-[CX3x0:3]=[NX2:4]	2
358	[cH1:1][c:2]([cH1])!@;-[CX3:3]=[NX2:4]	2
359	[cH0:1][c:2]([cH0])!@;-[CX3!r:3]=[NX2!r:4]	2
360	[a:1][c:2]!@;-[CX3:3]=[CX3H0:4]	2
361	[a:1][a:2]!@;-[CX3:3]=[CX3H2:4]	2
362	[a:1][a:2]!@;-[CX3:3]=[CX3H1:4]	2
363	[*:1][SX2:2]!@;-[SX2:3][*:4]	2
364	sp2-sp2	2
365	sp2-sp3	3
366	sp3-sp3	3
