# Supplementary material for: Bayesian optimization for conformer generation
Source: J Cheminform. 2019 May 21;11:32. doi: 10.1186/s13321-019-0354-7 (PMC6528340; doi:10.1186/s13321-019-0354-7)
Supplement: Supplementary file 2 — Additional file 2. List of molecules that excluded from analysis. [file 13321_2019_354_MOESM2_ESM.pdf]

Target	Number of rotatable bonds
astex_1p62	2
astex_1jd0	3
omegacsd_PAPPHI	3
omegacsd_PEXFED	3
omegapdb_1uwc	3
omegapdb_1d3g	3
omegacsd_YINDII	3
omegacsd_GALMOV	3
omegacsd_KAVLIC	3
astex_1n2j	3
omegacsd_WESZOJ	3
omegacsd_DUXYOK	3
omegacsd_YAHBOY	3
omegapdb_2aw1	3
omegacsd_MINTSA	3
omegacsd_CPIPLA	3
astex_1q1g	3
omegacsd_FABWUA10	3
omegapdb_1syh	3
omegacsd_CEVTUS	3
omegacsd_JUDLUP	3
omegacsd_GEKWAU	3
omegapdb_2evc	3
omegapdb_2flb	3
omegacsd_CMSMOC	3
omegacsd_DENBUT	4
omegacsd_PIKDIW	4
omegacsd_SIHVUA	4
omegacsd_WIKJOP	4
omegacsd_MATSTA10	4
omegapdb_1ofd	4
omegapdb_1y6q	4
omegacsd_MTBPNP	4
omegacsd_FOJZUZ	4
omegacsd_GINKUJ	4
omegacsd_YOPRAW	4
omegacsd_BAJZOB10	4
omegapdb_1qy5	4
omegacsd_BUTDID	4
omegacsd_GIHKAJ	4
omegacsd_VUGDEG	4
astex_1tow	4

Continued on next page

Target	Number of rotatable bonds
omegacsd_CAGXEN	4
omegacsd_KEBVES	4
omegacsd_ABHYTZ	4
omegapdb_2g8n	4
omegapdb_1gz8	4
omegacsd_TAPBZO	4
omegapdb_1yc1	4
omegacsd_FOLMEY	4
omegapdb_1uf7	4
omegacsd_CELDEC	4
omegacsd_DENDAC	4
omegacsd_CODYUP10	4
astex_1tz8	4
astex_1opk	4
omegacsd_EHMPYX10	4
omegapdb_1g6c	4
omegacsd_YUKKUK	4
omegacsd_DIGSIV	4
omegapdb_2cbs	4
omegacsd_DAWRAU	4
omegacsd_EITDZL	4
omegacsd_DIZREJ	4
omegapdb_1s8j	4
omegacsd_LETBUH	4
omegacsd_CMPEPI	4
omegapdb_1o3l	4
omegacsd_VOPDIN	4
omegacsd_GEJJUA	4
omegapdb_1rf6	4
omegacsd_LIKMEX	5
omegacsd_DIZWAK	5
omegacsd_YICPIJ	5
omegacsd_DEBBER	5
omegacsd_CETZOQ	5
omegacsd_VEYRAS	5
omegapdb_1v2k	5
omegacsd_NBPENC	5
omegacsd_WAVTIW	5
omegacsd_COKTAX	5
omegacsd_BTHYDX	5
omegacsd_WAMZAL	5
omegacsd_BHMPET	5

Continued on next page

Target	Number of rotatable bonds
omegacsd_ACPIXZ	5
omegacsd_VURTOR	5
omegapdb_1uf8	5
omegacsd_CBZHYX	5
omegacsd_LACPAG	5
omegacsd_FOLYIO	5
omegacsd_CLPNXA	5
omegacsd_YAHFAO	5
omegacsd_VIBWIM	5
omegapdb_1w9u	5
omegacsd_KICRIX	5
omegacsd_HEXFEV	5
omegacsd_YUYJEH	5
omegacsd_SILTUC	6
omegacsd_TAPSOS	6
omegacsd_DAFVUB	6
omegapdb_1h1s	6
omegacsd_BENPRL	6
omegacsd_SISYIC	6
omegacsd_CMANPQ	6
omegacsd_FLPNTX10	6
omegapdb_2gss	6
omegacsd_PMEPEN	6
astex_1v48	6
omegacsd_FOYLIO	6
omegacsd_SURREC	6
omegapdb_2j34	6
omegacsd_CFBPBI	6
omegacsd_ACENHT	6
omegacsd_FAHXIV	6
omegacsd_PEPHEX	6
omegacsd_FBPAZD	6
astex_1mzc	6
omegacsd_PMBSAN10	6
omegacsd_HALDOL	6
omegacsd_AOPCHY	6
omegacsd_FUJRUX	6
omegacsd_FANRER	6
omegapdb_6prc	6
omegacsd_BEKDIE	6
omegacsd_LEDWAS	6
