# Supplementary material for: Bayesian optimization for conformer generation
Source: J Cheminform. 2019 May 21;11:32. doi: 10.1186/s13321-019-0354-7 (PMC6528340; doi:10.1186/s13321-019-0354-7)
Supplement: Supplementary file 3 — Additional file 3. Figures for RMSD, TFD and maximum variation in energy. [file 13321_2019_354_MOESM3_ESM.pdf]

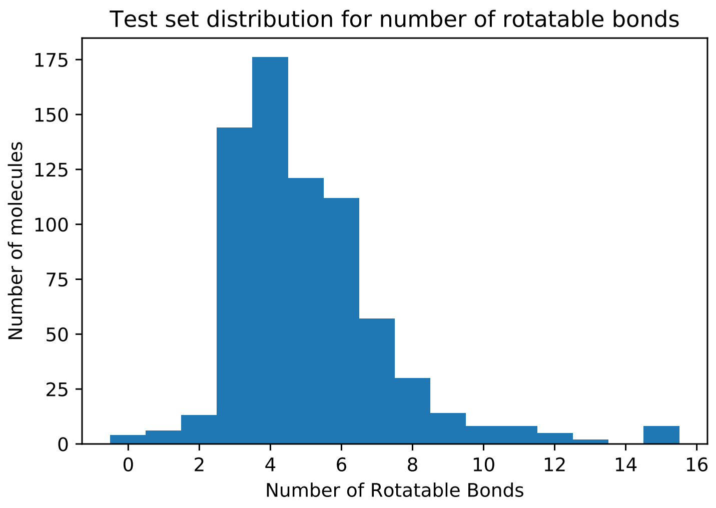

Figure 9 Test set distribution for number of rotatable bonds

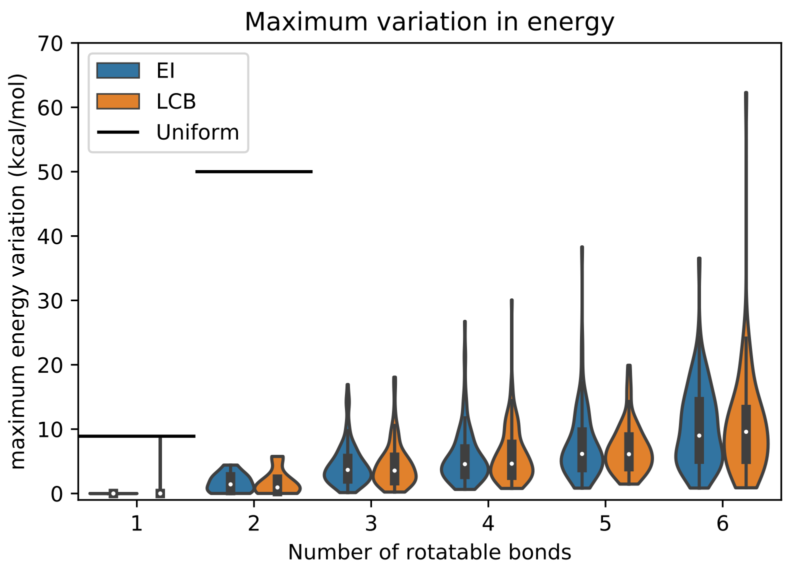

Figure 10 Maximum variation in energy by varying number of rotatable bonds in BOA: the distribution of the maximum difference between the lowest energy found from five independent trials and each individual trial, versus the number of rotatable bonds in each set of molecules. Note that 50 iterations are used for molecules with one to three rotatable bonds, while 100 iterations are used for molecules with four or more. The black horizontal line indicates the median of maximum energy variation in uniform random search. The median for molecules with three or more rotatable bonds are greater than 100 and reach approximately 6000 kcal/mol for molecules with six rotatable bonds, hence they are not shown in the figure.

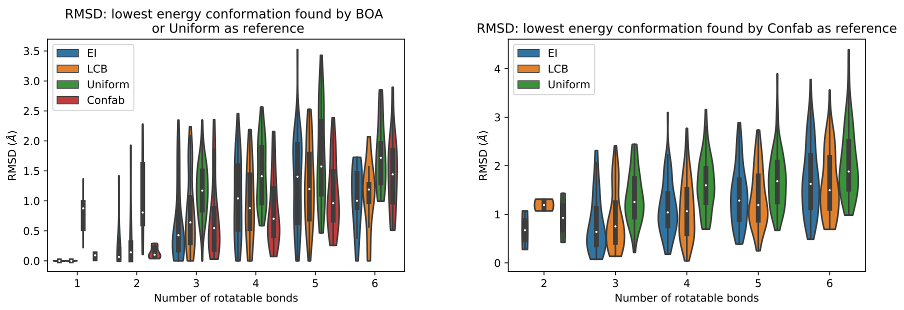

(a) Case 1 (b) Case 2

Figure 11 RMSD by varying number of rotatable bonds: (a) Case 1: lowest energy conformations found by either BOA or uniform random search is used as reference conformations. The small sample size for molecules with one and two rotatable bonds, 6 and 10 molecules, respectively, should be noted. (b) Case 2: lowest energy conformation found by Confab is used as reference conformations. Note there were only two molecules with two rotatable bonds. It can be seen that BOA with either EI or LCB outperformed uniform search. It is notable that BOA performed as well as Confab, despite BOA tending to use orders of magnitude fewer iterations than Confab.

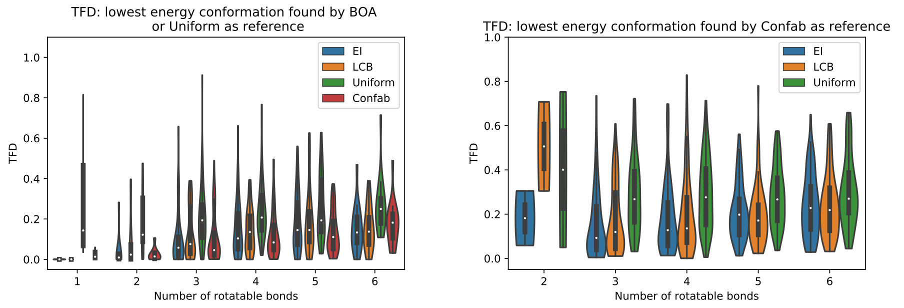

(a) Case 1 (b) Case 2

Figure 12 TFD by varying number of rotatable bonds: (a) Case 1: lowest energy conformations found by either BOA or uniform random search is used as reference conformations. It should also be noted that there is a small sample size for molecules with one and two rotatable bonds, 6 and 10 molecules, respectively. (b) Case 2: lowest energy conformations found by Confab is used as reference conformations. Note there were only two molecules with two rotatable bonds. It can be seen that BOA with either EI or LCB outperformed uniform search. As in Fig. 11, it is notable that BOA performed as well as Confab despite tending to use orders of magnitude fewer iterations.
