# Supplementary material for: Bayesian optimization for conformer generation
Source: J Cheminform. 2019 May 21;11:32. doi: 10.1186/s13321-019-0354-7 (PMC6528340; doi:10.1186/s13321-019-0354-7)
Supplement: Supplementary file 4 — Additional file 4. Tables of p-values for RMSD and TFD. [file 13321_2019_354_MOESM4_ESM.pdf]

Table 4 RMSD: Wilcoxon signed-rank test on each method pair. Case 1: the lowest energy conformations found by either BOA or uniform random search is used as reference conformation. Molecules with three or fewer rotatable bonds (N_{rot} : $1, 2, 3$) and molecules with five or more rotatable bonds (N_{rot} : $5, 6$) are grouped together respectively due to small sample size. Case 2: the lowest energy conformation found by Confab is used as reference conformation. Molecules with three or fewer rotatable bonds (N_{rot} : $1, 2, 3$) are grouped together due to small sample size. The p-values are rounded to 2 significant figures.

		N_{rot}			
Method Pairs	Case	1,2,3	4	5	6
EI-Uniform	1	$\mathbf{3.7 \times 10^{-10}}$	$\mathbf{6.5 \times 10^{-5}}$	$\mathbf{1.0 \times 10^{-3}}$	
EI-Confab	1	**0.04**	**0.04**	0.32	
EI-LCB	1	**0.03**	0.67	1.0	
LCB-Confab	1	0.64	0.24	0.73	
LCB-Uniform	1	$\mathbf{2.6 \times 10^{-7}}$	$\mathbf{6.1 \times 10^{-6}}$	$\mathbf{5.5 \times 10^{-5}}$	
Confab-Uniform	1	$\mathbf{3.1 \times 10^{-9}}$	$\mathbf{8.7 \times 10^{-8}}$	$\mathbf{1.5 \times 10^{-4}}$	
EI-LCB	2	0.03	0.66	0.96	0.76
EI-Uniform	2	$\mathbf{3.7 \times 10^{-10}}$	$\mathbf{6.5 \times 10^{-5}}$	0.06	$\mathbf{3.1 \times 10^{-3}}$
LCB-Uniform	2	$\mathbf{2.6 \times 10^{-7}}$	$\mathbf{6.1 \times 10^{-6}}$	$\mathbf{3.1 \times 10^{-3}}$	$\mathbf{4.8 \times 10^{-3}}$

Table 5 TFD: Wilcoxon signed-rank test on each method pair.Case 1: the lowest energy conformations found by either BOA or uniform random search is used as reference conformation. Molecules with three or fewer rotatable bonds (N_{rot} : $1, 2, 3$) and molecules with five or more rotatable bonds (N_{rot} : $5, 6$) are grouped together respectively due to small sample size. Case 2: the lowest energy conformation found by Confab is used as reference conformation. Molecules with three or fewer rotatable bonds (N_{rot} : $1, 2, 3$) are grouped together due to small sample size. The p-values are rounded to 2 significant figures.

		N_{rot}			
Method Pairs	Case	1,2,3	4	5	6
EI-Uniform	1	$\mathbf{1.3 \times 10^{-8}}$	$\mathbf{2.1 \times 10^{-5}}$	$\mathbf{3.1 \times 10^{-4}}$	
EI-Confab	1	0.70	0.14	0.14	
EI-LCB	1	0.07	0.94	0.74	
LCB-Confab	1	0.14	0.16	0.26	
LCB-Uniform	1	$\mathbf{1.0 \times 10^{-6}}$	$\mathbf{2.1 \times 10^{-5}}$	4.9×10^{-4}	
Confab-Uniform	1	$\mathbf{9.9 \times 10^{-10}}$	$\mathbf{5.0 \times 10^{-6}}$	$\mathbf{9.0 \times 10^{-6}}$	
EI-LCB	2	0.07	0.94	0.61	0.91
EI-Uniform	2	$\mathbf{1.3 \times 10^{-8}}$	$\mathbf{2.1 \times 10^{-5}}$	**0.01**	**0.01**
LCB-Uniform	2	$\mathbf{1.0 \times 10^{-6}}$	$\mathbf{2.1 \times 10^{-5}}$	$\mathbf{5.1 \times 10^{-3}}$	**0.04**
